# The triangular fibrocartilage complex on high-resolution 3 T MRI in healthy adolescents: the thin line between asymptomatic findings and pathology

**DOI:** 10.1007/s00256-021-03779-8

**Published:** 2021-04-17

**Authors:** Anne-Sophie van der Post, Sjoerd Jens, Frank F. Smithuis, Miryam C. Obdeijn, Roelof-Jan Oostra, Mario Maas

**Affiliations:** 1 Department of Radiology and Nuclear Medicine, Amsterdam Movement Sciences, Amsterdam UMC, University of Amsterdam, Meibergdreef 9, Amsterdam, The Netherlands; 2 Academic Center for Evidence-based Sports medicine (ACES), Amsterdam, The Netherlands; 3 Amsterdam Collaboration for Health and Safety in Sports (ACHSS), International Olympic Committee (IOC) Research Center AMC/VUmc, Amsterdam, The Netherlands; 4 Department of Radiology and Nuclear Medicine, Rijnstate Hospital, Arnhem, The Netherlands; 5 Department of Plastic, Reconstructive and Hand Surgery, Amsterdam Movement Sciences, Amsterdam UMC, University of Amsterdam, Meibergdreef 9, Amsterdam, The Netherlands; 6 Department of Medical Biology, Section Clinical Anatomy and Embryology, Amsterdam UMC, University of Amsterdam, Meibergdreef 9, Amsterdam, The Netherlands

**Keywords:** Triangular fibrocartilage, Adolescent, Magnetic resonance imaging, Wrist injuries

## Abstract

**Objective:**

The objective of the study is to provide a reference for morphology, homogeneity, and signal intensity of triangular fibrocartilage complex (TFCC) and TFCC-related MRI features in adolescents.

**Materials and methods:**

Prospectively collected data on asymptomatic participants aged 12–18 years, between June 2015 and November 2017, were retrospectively analyzed. A radiograph was performed in all participants to determine skeletal age and ulnar variance. A 3-T MRI followed to assess TFCC components and TFCC-related features. A standardized scoring form, based on MRI definitions used in literature on adults, was used for individual assessment of all participants by four observers. Results per item were expressed as frequencies (percentages) of observations by all observers for all participants combined (*n* = 92). Inter-observer agreement was determined by the unweighted Fleiss’ kappa with 95% confidence intervals (95% CI).

**Results:**

The cohort consisted of 23 asymptomatic adolescents (12 girls and 11 boys). Median age was 13.5 years (range 12.0–17.0). Median ulnar variance was −0.7 mm (range − 2.7–1.4). Median triangular fibrocartilage (TFC) thickness was 1.4 mm (range 0.1–2.9). Diffuse increased TFC signal intensity not reaching the articular surface was observed in 30 (33%) observations and a vertical linear increased signal intensity with TFC discontinuation in 19 (20%) observations. Discontinuation between the volar radioulnar ligament and the TFC in the sagittal plane was seen in 23 (25%) observations. The extensor carpi ulnaris was completely dislocated in 10 (11%) observations, more frequent in supinated wrists (*p* = 0.031). Inter-observer agreement ranged from poor to fair for scoring items on the individual TFCC components.

**Conclusion:**

MRI findings, whether normal variation or asymptomatic abnormality, can be observed in TFCC and TFCC-related features of asymptomatic adolescents. The rather low inter-observer agreement underscores the challenges in interpreting these small structures on MRI. This should be taken into consideration when interpreting clinical MRIs and deciding upon arthroscopy.

**Supplementary Information:**

The online version contains supplementary material available at 10.1007/s00256-021-03779-8.

## Introduction

The triangular fibrocartilage complex (TFCC) is a complex of fibro-cartilaginous and ligamentous structures, located between the distal ulna and carpal bones, facilitating distal radioulnar joint (DRUJ) stabilization, carpal stabilization, and axial load transmission [1]. TFCC injury can be sustained by trauma typically due to a fall on the outstretched hand, which is common in adults and children, yet it can also result from degeneration due to repetitive forces on the wrist in young athletes and adults [2, 3]. It is generally considered a common cause of ulnar-sided wrist pain [3, 4]. However, ulnar-sided wrist pain comprises a challenging diagnostic process due to the wide differential diagnosis for this small anatomic region [5].

After physical examination, the first diagnostic step in ulnar-sided wrist pain is a standardized radiograph. This can show TFCC injury associated abnormalities such as ulnar styloid process fractures, positive ulnar variance, or degenerative changes [4, 6]. Magnetic resonance imaging (MRI) can be performed when a clinical suspicion of TFCC injury remains. Arthroscopy, the diagnostic reference standard, is performed only when prompted by physical examination combined with MRI abnormalities [7].

However, clinicians should be aware of the high prevalence of TFCC “abnormalities” on MRI in the asymptomatic population ranging from 19% in populations under 30 years to 64% in populations over 70 years [8, 9]. Additionally, MRI has remained controversial due to significant variations in diagnostic accuracy [10]. This can be explained by differences in observer experience and MRI quality as well as by the wide variation in definitions and criteria for diagnosing TFCC injury [8, 11–13].

Once thought rarely to occur in pediatric patients, arthroscopy recently showed TFCC tears in 48.5–80.5% of pediatric patients with persistent wrist pain [14–16]. Despite the absence of knowledge regarding TFCC features and its normal variation on MRI in children and adolescents, an increasing number of articles on surgical techniques and outcomes for TFCC injury have been published since [17–21]. We are not aware of any studies on TFCC features on MRI in adolescents and hypothesize that TFCC abnormalities are also prevalent in the asymptomatic pediatric population. Therefore, we assessed MRIs of healthy asymptomatic adolescents in order to obtain a reference for morphology, homogeneity, and signal intensity of the individual TFCC components and TFCC-related features. This will assist in addressing MRI findings of the TFCC in patients with ulnar-sided wrist pain as either pathological or non-pathological and accordingly improve TFCC injury diagnosis.

## Materials and methods

This observational cross-sectional study is a retrospective analysis of prospectively collected study data from adolescents involved in the Physeal MRI study that selected gymnasts with wrist pain, asymptomatic gymnasts, and healthy participants aged 12–18 years in Amsterdam University Medical Center, Location AMC, from June 2015 to November 2017 [22]. The current study only included the healthy participants. The study was performed in accordance with the Declaration of Helsinki and approved by the institution’s Medical Review Ethics Committee (reference no. 2014_382). Written informed consent was given by each participant as well as the parent or legal guardian.

### Population

Healthy girls and boys aged 12–18 years without wrist pain over the last 6 months were included. Participants were not eligible if they had participated in gymnastics or performed wrist-loading sports over two times a week, had fused distal radial physes, were diagnosed with growth disturbance or musculoskeletal diseases, or had a history of wrist fracture, surgery, or infection.

### Imaging

All participants underwent a radiograph followed by MRI of one wrist. Conventional posterior-anterior radiographs were obtained in 90° shoulder abduction, 90° elbow flexion, and neutral forearm position (focus-detector distance 1.30 m). Skeletal age was determined using the validated BoneXpert software (v2.0.1.3; Visiana, Holte, Denmark). Ulnar variance was assessed on the radiographs by one specialized musculoskeletal radiologist (SJ) using the recommended perpendicular method [23]. Negative ulnar variance indicates a relatively shorter ulna. MRIs were obtained with a 3-T MRI scanner (Ingenia, Philips Healthcare, Best, The Netherlands) in a feet-first, supine position with the wrist placed neutral, alongside the body, using a dedicated wrist coil (eight channel, receive-only). The analyzed MRI sequences included turbo spin-echo (TSE) proton-density (PD) weighted sequences in three planes without fat saturation (1500–2000 ms repetition time (TR), 20 ms echo time (TE), 90° flip angle, 2.5–1.5 mm slice thickness, 0.30 × 0.30 mm spatial resolution, 4:06–4:39 min scan time), a TSE PD weighted coronal sequence with fat saturation by spectral attenuated inversion recovery (SPAIR) (2000 ms TR, 30 ms TE, 90° flip angle, 2.5 mm slice thickness, 0.30 × 0.32 mm spatial resolution, 4:12 min scan time), and a TSE T2 weighted axial sequence with fat saturation by SPAIR (3001 ms TR, 60 ms TE, 90° flip angle, 2.5 mm slice thickness, 0.30 × 0.36 mm spatial resolution, 4:58 min scan time).

### Standardized scoring form

A standardized scoring form focusing on observation of morphology, homogeneity, and signal intensity and explicitly not on detecting pathology was constructed by a physician experienced in research on musculoskeletal imaging (AP). Due to the lack of a consensus reference standard regarding the individual TFCC components, the items were based on MRI definitions used by Zhan et al. on adults, i.e., triangular fibrocartilage (TFC), dorsal and volar radioulnar ligaments (RUL), meniscus homolog, extensor carpi ulnaris (ECU) tendon sheath, ulnocarpal ligaments (ulnolunate and ulnotriquetral), and ulnar collateral ligament [24]. Images from trial cases that were not included in the present study (i.e., symptomatic gymnasts) were provided as illustrative examples in the scoring form (see supplementary material).

First, scoring items were adjusted, added, and deleted based on clinical relevance and feasibility in several rounds of consensus meetings with a single-center expert group of three musculoskeletal radiologists (MM, FS en SJ with 26, 5 and 1 years of experience, respectively) and one hand surgeon (MO with 15 years of experience). Items on ulnocarpal ligaments and the ulnar collateral ligament were deleted due to lack of clinical relevance and controversy over their existence [25]. The recommended sequences and planes for assessment were determined in consensus and stated per item. The final score form, as provided by the supplementary material, was piloted in several trial cases for calibration prior to scoring.

Then, each member of the expert group individually assessed the blinded MRI scans in a randomized order on a diagnostic PC workstation with a high-resolution monitor using the IMPAX software version 6.6.1.4024 (AGFA HealthCare N.V., Mortsel, Belgium). In order to prevent response fatigue, observers were allowed to assess only three consecutive MRIs followed by a mandatory break. Each observer was blinded for other observers’ scoring results and observers were aware that subjects were asymptomatic. Windowing, zooming, and scrolling through the images was allowed.

### Statistical analysis

Patient characteristics and scoring form observations were entered into Castor Electronic Data Capture (Ciwit BV, Amsterdam, The Netherlands, 2018) and directly imported for analysis in RStudio (RStudio, Inc., Boston). Descriptive statistics and TFC thickness were illustrated as medians with ranges. TFC thickness measurements that were not possible due to disc disruption were excluded from this analysis. An intraclass correlation coefficient (ICC) for inter-observer agreement on TFC thickness was calculated using a two-way random-effects (agreement, single measures) model. For each MRI characteristic, categorical variables scored by all observers for all participants were combined and expressed in frequencies of observations with percentages (*n* = 92). Inter-observer agreement was determined by the unweighted Fleiss’ kappa with 95% confidence intervals (95% CI). All correlation coefficients were interpreted as poor (≤ 0.20), fair (0.21–0.40), moderate (0.41–0.60), substantial (0.61–0.80), or excellent (0.81–1.00) agreement [26]. Differences were calculated with a Mann-Whitney *U* test. *P* values <0.05 were considered statistically significant.

## Results

### Patient characteristics

The study cohort consisted of 23 healthy adolescents and their demographic information is depicted in Table 1. One boy was excluded due to insufficient image quality based on motion artifacts.

**Table 1:** Participant demographics

Items	Number (percentage)
Participants	23 (100)
Sex (girls/boys)	12 (52)/11 (48)
Wrist (right/left)	13 (57)/10 (43)
	Median (range)
Calendar age (years)	13.5 (12.0–17.0)
Skeletal age (years)	13.4 (11.3–17.6)
Ulnar variance (millimeters)	−0.7 (−2.7–1.4)

### TFCC components

All results for MRI characteristics of TFCC components are shown in Table 2.

**Table 2:** MRI characteristics scored by all observers in frequencies of observations for all participants by all observers combined with percentages calculated from the total amount of observations (*n* = 92)

Scoring item	MRI characteristics	Frequency (%)	Kappa (95% CI)
Triangular fibrocartilage			
Coronal morphology	Slightly radially tilted bowtie	46 (50)	0.33 (0.20–0.46)
	Shorter, thicker and more horizontal	34 (37)	
	Thinner and more stretched	12 (13)	
Coronal homogeneity	Homogeneous hypo-intense	43 (47)	0.13 (0.16–0.24)
	Diffuse increased SI	30 (33)	
	Vertical linear increased SI	14 (15)	
	Other	5 (5)	
Sagittal morphology	Symmetrical biconcave	48 (52)	0.19 (0.20–0.53)
	Dorsal thicker than volar	41 (45)	
	Volar thicker than dorsal	3 (3)	
Dorsal radioulnar ligament			
Sagittal morphology	Continuous with TFC SI	74 (80)	0.20 (0.07–0.32)
	Discontinuous with TFC SI	9 (10)	
	Not able to assess	9 (10)	
Axial fiber continuity	Continuous fibers	77 (84)	0.06 (−0.07–0.19)
	Fiber discontinuation	5 (5)	
	Not able to assess	10 (11)	
Volar radioulnar ligament			
Sagittal morphology	Continuous with TFC SI	55 (60)	0.23 (0.11–0.36)
	Discontinuous with TFC SI	23 (25)	
	Not able to assess	14 (15)	
Axial fiber continuity	Continuous fibers	82 (89)	−0.04 (−0.19–0.12)
	Fiber discontinuation	1 (1)	
	Not able to assess	9 (10)	
Proximal lamina			
Coronal homogeneity	Homogeneous hypo-intense	51 (55)	0.18 (0.04–0.32)
	Diffuse lamination	33 (36)	
	Not able to assess	8 (9)	
Distal lamina			
Coronal homogeneity	Homogeneous hypo-intense	44 (48)	0.13 (−0.01–0.26)
	Diffuse lamination	39 (42)	
	Not able to assess	9 (10)	
Ligamentum subcruentum			
Coronal morphology	Hyper-intense signal between lamina	74 (80)	0.22 (0.05–0.38)
	Not visible	18 (20)	
Meniscus homolog			
Coronal morphology	Diffuse hypo-intensity	52 (57)	0.04 (−0.08–0.16)
	Clearly delineated hypo-intensity	24 (26)	
	Not visible	16 (17)	
Wrist position			
Axial position	Supination	47 (51)	0.62 (0.53–0.84)
	Neutral	42 (46)	
	Pronation	3 (3)	
Extensor carpi ulnaris tendon			
Axial position	Completely within groove	51 (55)	0.59 (0.46–0.72)
	Partially within groove	31 (34)	
	Completely outside groove	10 (11)	
Axial peri-tendinous SI	Hypo-intense/intermediate	48 (52)	0.22 (0.05–0.38)
	Focal increased	44 (48)	
Axial intra-tendinous SI	Homogeneous hypo-intense	49 (53)	0.29 (0.12–0.45)
	Focal or linear increased	43 (47)	
Joint effusion			
Prestyloid recess	Conical shaped	37 (40)	0.31 (0.21–0.42)
	Tubular shaped	17 (18)	
	Saccular shaped	10 (11)	
	Not visible	28 (30)	
DRUJ effusion	Absent	22 (24)	0.30 (0.18–0.43)
	Small amount	57 (61)	
	Substantial amount	12 (14)	
PTJ effusion	Absent	22 (24)	0.43 (0.31–0.55)
	Small amount	44 (48)	
	Substantial amount	26 (28)	
Ulnar-sided cysts	Absent	81 (88)	0.36 (0.23–0.50)
	Present volar	8 (9)	
	Present dorsal	3 (3)	

*DRUJ* distal radioulnar joint, *PTJ* pisotriquetral joint, *TFC* triangular fibrocartilage, *SI* signal intensity

### Triangular fibrocartilage (TFC)

**Fig. 1:**
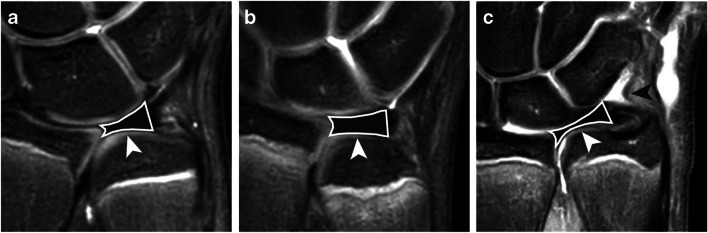
Coronal proton-density weighted fat-saturated MRI showing TFC morphology delineated in white and indicated by the white arrowheads **a** as a slightly radially tilted asymmetrical bowtie, **b** as a shorter, thicker and more horizontal structure, and **c** as a thinner and more stretched structure. Image **c** also shows the meniscus homolog morphology as a diffuse triangular shaped hypo-intensity (black arrowhead) directly lateral from the prestyloid recess

Median TFC thickness was 1.4 mm (range 0.1–2.9). Agreement between observers for measuring TFC thickness was moderate (ICC = 0.60, 95% confidence interval = 0.40–0.77). In the coronal plane, TFC morphology was identified as a slightly radially tilted asymmetrical bowtie in 46 (50%) observations, as a shorter, thicker, and more horizontal structure in 34 (37%) and as a thinner and more stretched structure in only 12 (13%) (Fig. 1). We found no statistical difference in wrist position (*p =* 0.26*)* or ulnar variance (*p =* 0.17*)* for wrists where a thinner and more stretched TFC configuration was observed. In the sagittal plane, TFC morphology was scored as a symmetrical biconcave disc in 48 (52%) and as an asymmetrical disc being thicker at the dorsal side than at the volar side in 41 (45%) observations. Inter-observer agreement for scoring coronal morphology was fair (ICC = 0.33, 95% CI = 0.20–0.46) and for scoring sagittal morphology was poor (ICC = 0.19, 95% CI = 0.20–0.53).

**Fig. 2:**
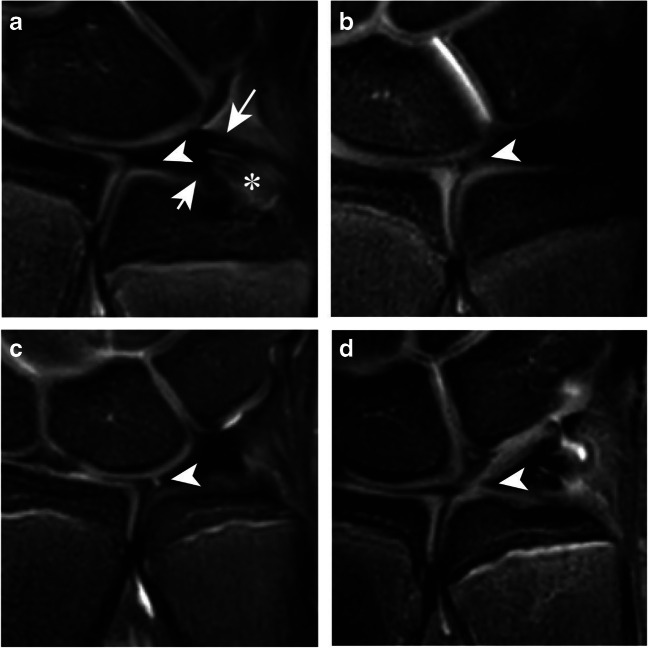
Coronal proton-density weighted fat-saturated MRI showing TFCs (indicated with arrowheads) with **a** hypo-intense signal intensity, **b** diffuse hyper-intense signal intensity not extending through the articular surface, **c** vertical linear hyper-intense signal intensity with discontinuation of the disc, and **d** a broader variation on this vertical line. Image **a** also shows the proximal (short arrow) and distal lamina (long arrow) as homogeneous hypo-intense bands, separated by the ligamentum subcruentum shown as a hyper-intense signal intensity (asterisk)

TFC homogeneity in the coronal plane was scored as a homogeneous hypo-intense signal intensity in 43 (47%) and as a diffuse increased signal intensity not reaching the articular surface in 30 (33%) (Fig. 2). In 14 (15%) observations regarding TFC homogeneity, a vertical linear increased signal intensity with discontinuation of the TFC was identified. Other types of homogeneity that were scored included slight morphological variations on this vertical line of increased signal intensity. Inter-observer agreement for coronal homogeneity was poor (ICC = 0.13, 95% CI = 0.16–0.24).

### Dorsal and volar radioulnar ligaments (RUL)

**Fig. 3:**
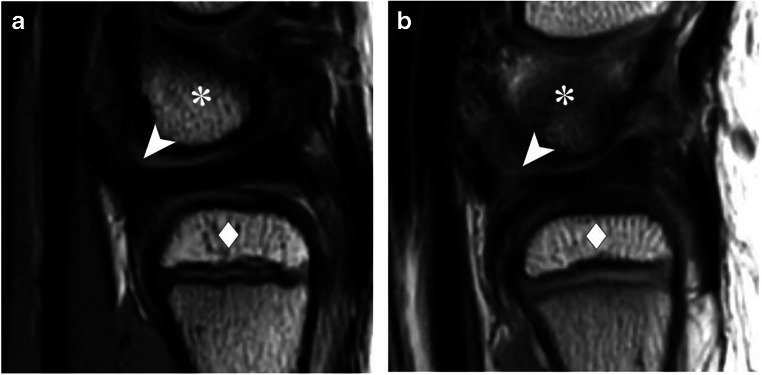
Sagittal proton-density weighted MRI showing the volar RUL as a structure that is **a** continuous and indiscernible with the TFC (arrowhead) and **b** discontinuous with the TFC (arrowhead) in between the ulnar head (diamonds) and lunate bone (asterisks)

In the sagittal plane, the dorsal and volar RUL were scored as continuous and indiscernible structures with the TFC in 74 (80%) and 55 (60%) observations, respectively, while the volar RUL signal intensity appeared to be discontinuous with the TFC in 23 (25%) observations (Fig. 3). The inter-observer agreement was fair (ICC = 0.23, 95% CI = 0.11–0.36) for volar RUL and poor (ICC = 0.20, 95% CI = 0.07–0.32) for dorsal RUL sagittal morphology. Axial RUL fiber continuity was scored as continuous in 77 (84%) observations of the dorsal RUL and 82 (89%) of the volar RUL. In 5 (5%) observations, the dorsal RUL showed fiber discontinuation, for the volar RUL fiber discontinuation was seen in 1 (1%) observation. For this item, inter-observer agreement regarding the dorsal and volar RUL was poor (ICC = 0.06, 95% CI = −0.07–0.19 and ICC = −0.04, 95% CI = −0.19–0.12, respectively).

### Proximal (deep) and distal (superficial) lamina

Homogeneity of the proximal and distal lamina on the coronal view was scored as homogeneous hypo-intense in 51 (55%) (Fig. 2a) and 44 (48%) observations, respectively. In 33 (36%) of the proximal and 39 (42%) of the distal lamina, a diffuse lamination with hyper-intense signals was seen. Inter-rater agreement for both proximal and distal lamina coronal homogeneity was poor (ICC = 0.18, 95% CI = 0.04–0.32 and ICC = 0.13, 95% CI = −0.01–0.26, respectively).

### Ligamentum subcruentum

Coronal morphology of the ligamentum subcruentum was identified as a hyper-intense signal intensity in between the proximal and distal lamina in 74 (80%) observations (see Fig. 2a). In 18 (20%) observations, this structure was not clearly visible. Agreement for this item was fair (ICC = 0.22, 95% CI = 0.05–0.38).

### Meniscus homolog (MH)

Meniscus homolog morphology on the coronal view was scored as a diffuse triangular shaped hypo-intensity directly ulnar from the prestyloid recess in 52 (57%) observations (Fig. 1c). In 24 (26%) observations, this triangular-shaped hypo-intensity was clearly demarcated. The meniscus homolog was not discernable and therefore scored as not visible in 16 (17%) observations. Inter-observer agreement was poor (ICC = 0.04, 95% CI = −0.08–0.16).

### TFCC-related features

The results of additional TFCC-related MRI characteristics of the ulnar side of the wrist are also shown in Table 2.

### Wrist and ECU tendon position

**Fig. 4:**
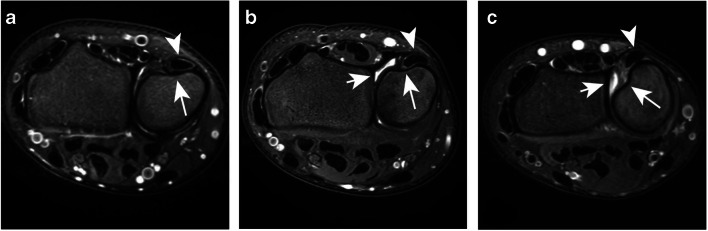
Axial T2 weighted fat-saturated MRI showing the ECU tendon (arrowheads) position **a** completely within, **b** partially within, and **c** completely outside its groove (long arrows). All three wrist positions were scored as a supinated wrist position with the ulnar head in dorsal rotation. Image **b** and **c** also show a substantial amount of DRUJ effusion (short arrows)

Neutral wrist position based on the axial plane was seen in 42 (46%) observations. The wrist was defined as supinated in 47 (51%) observations and pronated in 3 (3%). The ECU tendon position was scored as completely positioned within its groove in 51 (55%), as partially within its groove in 31 (34%), and as completely dislocated out of its groove in 10 (11%) observations (Fig. 4). The distribution of ECU tendon positions significantly differed between a supinated and a non-supinated wrist position (*p* = 0.031, Table 3). Inter-rater agreement for wrist and ECU tendon position was, respectively, substantial (ICC = 0.62, 95% CI = 0.53–0.84) and moderate (ICC = 0.59, 95% CI = 0.46–0.72).

**Table 3:** Contingency table for the position of the extensor carpi ulnaris tendon regarding the ulnar groove and wrist position during MRI expressed in frequencies with percentages

	Position extensor carpi ulnaris tendon regarding ulnar groove			
	Completely within	Partially within	Completely outside	Total
Supination	22 (23.9)	16 (17.4)	9 (9.7)	47 (51.0)
Neutral position	26 (28.3)	15 (16.3)	1 (1.1)	42 (45.7)
Pronation	3 (3.3)	0 (0.0)	0 (0.0)	3 (3.3)
Total	51 (55.4)	31 (33.7)	10 (10.9)	92 (100.0)

### ECU tendon signal intensity

The signal intensity surrounding and inside the ECU tendon in the axial plane was assessed on the slices in between the proximal base of the ECU groove and the distal border of the extensor retinaculum. The signal intensity was scored as hypo-intense to intermediate in 48 (52%) and 49 (53%) observations, respectively. In 44 (48%) of the peri-tendinous and 43 (47%) of the intra-tendinous signal intensities, focal or linear increased signal intensity was observed. Peri-tendinous increased signal was located proximal from the styloid process in 34 (77%) observations. Intra-tendinous increased signal was located at the styloid level in 29 (67%). Inter-observer agreement for scoring ECU tendon signal intensity was fair for peri-tendinous (ICC = 0.22, 95% CI = 0.05–0.38) as well as intra-tendinous (ICC = 0.29, 95% CI = 0.12–0.45) signal intensity.

### Joint effusion

**Fig. 5:**
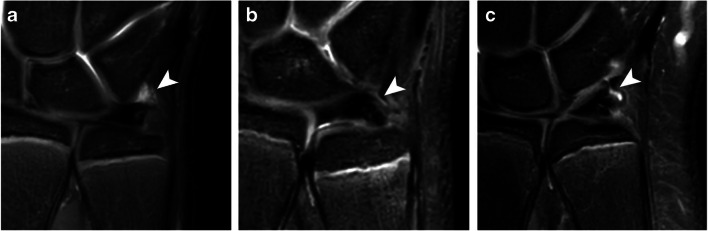
Coronal proton-density weighted fat-saturated MRI showing the **a** conical shaped prestyloid recess (arrowhead), **b** tubular shaped prestyloid recess (arrowhead), and **c** saccular shaped prestyloid recess (arrowhead)

Coronal morphology of the prestyloid recess was scored as a conical shape in 37 (40%) observations, a tubular shape in 17 (18%), and a saccular shape in 10 (11%) (Fig. 5). In 23 (30%) assessments, the prestyloid recess itself was not visible. A fair (ICC = 0.31, 95% CI = 0.21–0.42) inter-observer agreement for this item was found. A small amount of distal radioulnar joint (DRUJ) and pisotriquetral joint (PTJ) effusion was present according to 37 (40%) and 44 (48%) observations, respectively. In 12 (14%) and 26 (28%) observations, a substantial amount of DRUJ and PTJ effusion was identified, respectively. Inter-observer agreement was fair (ICC = 0.30, 95% CI = 0.18–0.43) for DRUJ effusion and moderate (ICC = 0.43, 95% CI = 0.31–0.55) for PTJ effusion. Additional ulnar-sided cysts were identified at the volar side in 8 (9%) and on the dorsal side in 3 (3%) observations, with a fair (ICC = 0.36, 95% CI = 0.23–0.50) inter-observer agreement.

### Other relevant findings

Two observers noticed physeal edema in the distal radius and ulna from one participant and three observers noticed a type two lunate bone with bone marrow edema in one participant. Besides several radial and carpal ganglion cysts, no other relevant findings were observed.

## Discussion

Knowledge regarding TFCC characteristics on MRI in adolescents is lacking in the literature. In this study, a wide range in variation of individual TFCC components and TFCC-related features on MRI was observed in healthy asymptomatic adolescents. Generally, the TFC appeared as a slightly radially tilted bowtie with homogeneous hypo-intense signal intensity in coronal view and as a symmetrical biconcave disc in the sagittal view. The RULs were mostly continuous with TFC. The proximal and distal lamina was homogeneously hypo-intense or diffusely laminated and separated by a hyper-intense ligamentum subcruentum. The meniscus homolog was a diffuse triangular shaped hypo-intensity lateral from the prestyloid recess.

The coronal morphology of the disc was observed as thinner and more stretched in 13% (ICC = 0.33, 95% CI = 0.20–0.46) of the observations. Literature has suggested that this stretched shape of the disc is associated with a positive ulnar variance [27]. Yet, the present study did not find a statistical difference in ulnar variance when the stretched morphology was scored. Probably, this can be explained by the small numbers of observations of this stretched shape (*n* = 12). Neither did we find a statistical difference in wrist position, which is in accordance with a previous observation that shape of the disc did not change during pro- or supination [28].

A diffuse increased signal intensity within the disc, considered a sign of TFC degeneration in adults (Palmer 2A injury), was seen in 33% (ICC = 0.13, 95% CI = 0.16–0.24) of the observations [29]. Since the young participants did not perform wrist-loading sports more than twice a week, this highly unlikely represents degeneration. Also, a vertical linear increased signal intensity of the TFC with discontinuation of the disc, currently interpreted as a traumatic TFC perforation (Palmer 1A injury), was seen in 20% of observations [24].

It is known that newborns have higher percentages of vascularized peripheral TFC margins than adults [30]. On MRI of the knee in children, similar signal intensities within the menisci that tended to decrease with age were found and authors suggest they might reflect normal increased vascularization [31]. Since TFC vascularization appears to resemble menisci, this could be an explanation for both observed TFC signal intensity changes. However, it is difficult to distinguish the vertical hyper-intense lines that appear as perforations from vascularity in this study.

An early study on TFC perforations that is still frequently referred to did not observe perforations in the first two decades of life [32]. Even though a subsequent study on gross anatomy of fetal and newborn cadaveric wrists reported TFC perforations in 27 out of 120 (22.5%) wrists, this type of “injury” is still used as indication for arthroscopic debridement in pediatric patients with persistent wrist pain [33, 34]. We found a comparable incidence of signal intensities imposing as TFC perforations, yet future studies should demonstrate the anatomical substrate of these linear signal intensity changes.

With the recent development of MRI techniques, additional TFCC abnormalities can be visualized [24]. Discontinuation between the dorsal and volar edges of the TFC with the joint capsule is currently suggested as a pathological capsular detachment and as an additional type of Palmer injury [24]. In the present study, a discontinuation of the volar RUL with the TFC due to a linear hyper-intense signal intensity was found in 23 (25%) observations mimicking this capsular detachment. In the axial plane, however, only one observer found fiber discontinuation of the volar radioulnar ligament. Therefore, we can conclude that this observation rather reflected a volume averaging artifact than a true fiber discontinuation. This stresses the importance of using both imaging planes when assessing the radioulnar ligaments.

We observed a high prevalence of ECU tendon (sub)luxation (44%), which is even higher than reported by a recent study in 123 asymptomatic adults that observed 30% of ECU tendon (sub)luxation [35]. Mild ECU tendon subluxation is frequently observed in the supinated wrist; however, rupture of the ECU subsheath is considered to cause greater subluxation in supination [36]. In the present study, 51% of the wrists were scanned in a supinated position. Additionally, 90% of the wrists with complete ECU tendon luxation were identified as supinated. Therefore, in case of a subluxation or complete luxation of the ECU tendon in adolescents, this should not be associated with ECU tendon subsheath injury if scanned in a supinated position.

The high rate of supination in the present study is a limitation, especially since a neutral wrist position is key in assessing the dorsal and volar RUL [5]. In line with current clinical practice, the general adult wrist coils were used. Despite careful neutral positioning prior to scanning, this probably enabled increased moving space after placement in neutral position. We therefore recommend additional elbow support and focus on fixation in MRI wrists involving adolescents. Another limitation was the fact that observers were aware of the asymptomatic character of the participants. However, the effect of possible interpretation bias was limited since we intended to report imaging observations rather than interpretations on pathology.

Despite multiple consensus meetings and a calibration session, the inter-observer agreement in TFCC assessment was rather low. However, due to our diligence in designing the scoring items, we do not consider this to reflect a limited methodology, but rather the personal interpretation that TFCC assessment in clinical practice is largely subject to [10, 13]. We believe that variation in structural terminology of TFCC components across studies is a major complicating factor and agree that clinical practice would benefit from consensus on TFCC definitions and abnormality criteria [8]. Especially, since wrist MRIs change the suspected clinical diagnosis in 46% of predominantly surgical referrals in pediatric patients under 18 years and even lead to a change in therapeutic strategy in 86% [37]. This, in combination with a high rate of asymptomatic MRI findings of the TFCC, bears the risk of unnecessary wrist arthroscopy and emphasizes the importance of multidisciplinary decision-making.

In conclusion, the present study showed that asymptomatic MRI findings, whether normal variation or asymptomatic abnormalities, can be observed in individual TFCC components and TFCC-related features of healthy asymptomatic adolescents. MRI findings that can be interpreted as abnormalities such as TFC perforations, TFC degeneration, pathological capsular detachment of the volar RUL, and ECU tendon (sub)luxation indicating ECU subsheath injury were observed. It is important to consider these asymptomatic findings when interpreting MRIs of the wrist in symptomatic pediatric patients and deciding upon arthroscopic treatment.

## Supplementary Information

ESM 1 (DOCX 3.62 mb)
