# Supplementary material for: The triangular fibrocartilage complex on high-resolution 3 T MRI in healthy adolescents: the thin line between asymptomatic findings and pathology
Source: Skeletal Radiol. 2021 Apr 17;50(11):2195–204. doi: 10.1007/s00256-021-03779-8 (PMC8449761; doi:10.1007/s00256-021-03779-8)
Supplement: Supplementary file 1 — (DOCX 3.62 mb) [file 256_2021_3779_MOESM1_ESM.docx]

| **Supplementary Material.** Score form used for triangular fibrocartilage complex assessment in healthy asymptomatic adolescents with illustrative images from young symptomatic gymnasts that were not included in the present study | | | | | |
| --- | --- | --- | --- | --- | --- |
| **A** | **WRIST POSITIONING** | |  | | |
| 1.1 | **Ulnar prestyloid recess position**  *Axial PD* | 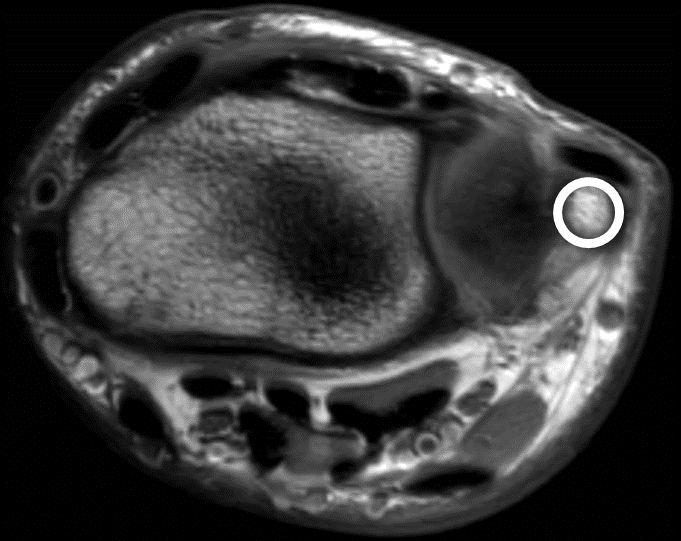  □ Neutral | | 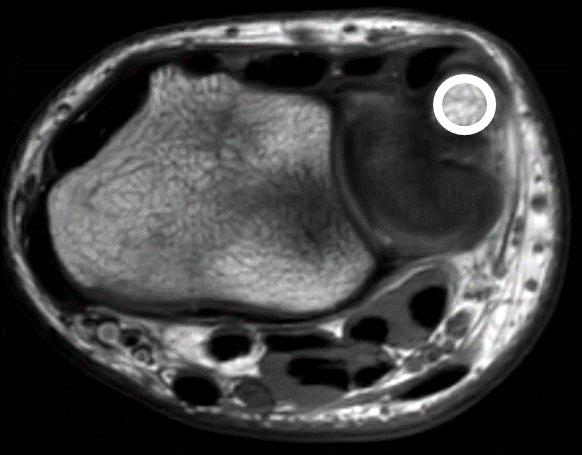  □ Dorsal rotation (supinated) | 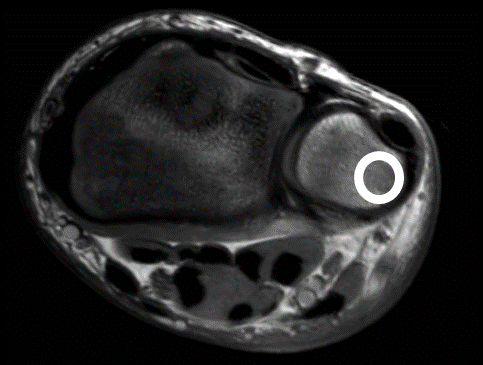  □ Volar rotation (pronated) |
| **B** | **TRIANGULAR FIBROCARTILAGE (TFC)** | |  | | |
| 1.1 | **TFC morphology – On the sagittal slice where the TFC is at its thinnest**  *Sagittal PD* | 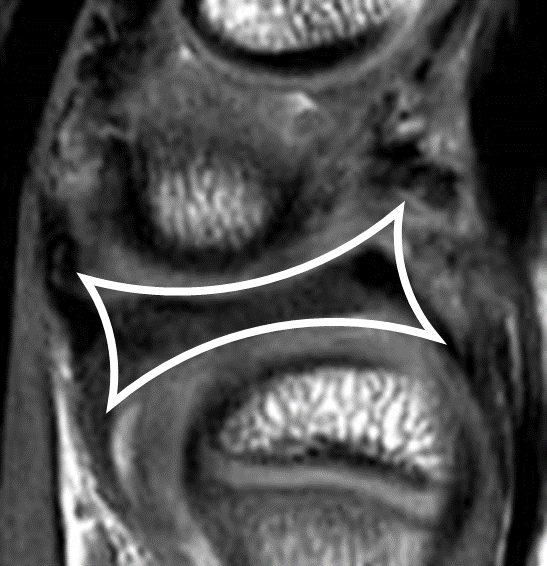  □ Symmetrical biconcave disc | | 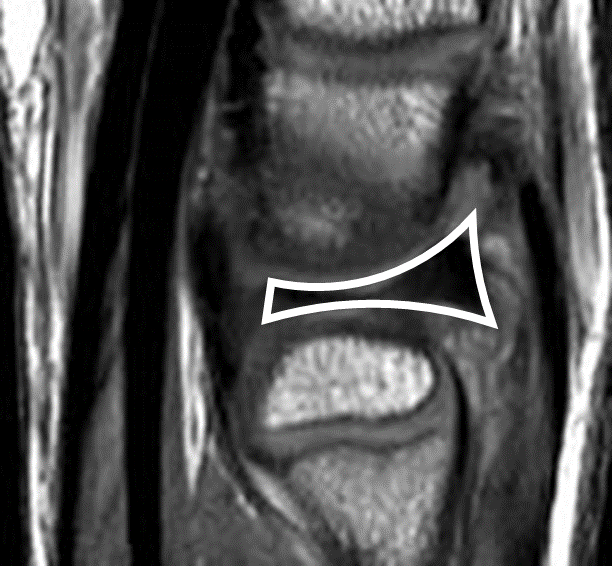  □ Biconcave disc dorsal thicker than volar | 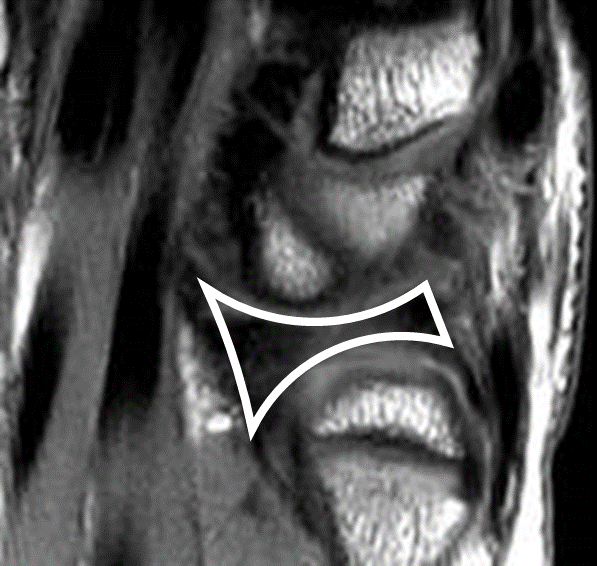  □ Biconcave disc volar thicker than dorsal |

| 1.2 | **TFC morphology – On the mid-coronal slice where the TFC is at its thinnest**  *Coronal PD and PD SPAIR* | 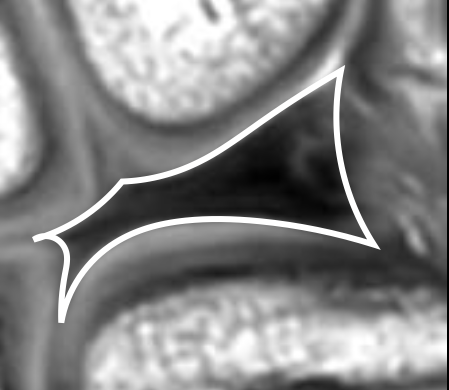  □ Slightly radial tilted asymmetrical bowtie | | 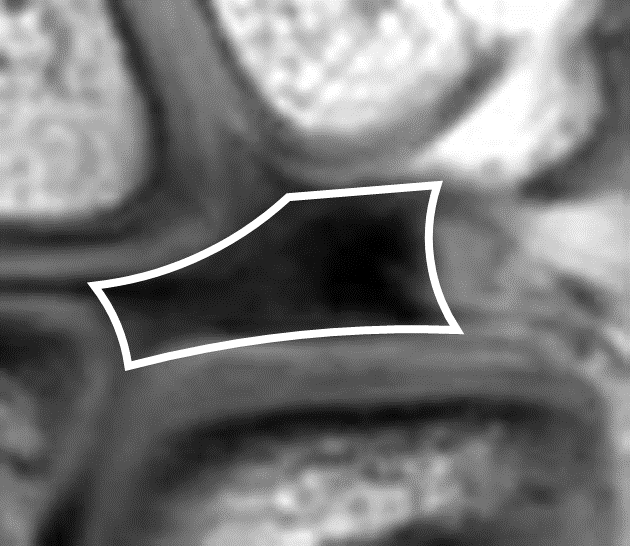  □ Shorter, thicker and more horizontal structure | | 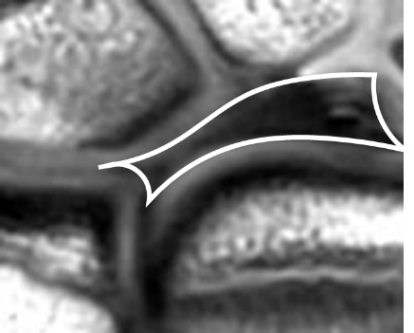  □ Thinner and more stretched structure | |
| --- | --- | --- | --- | --- | --- | --- | --- |
| 1.3 | **TFC thickness – On the mid-coronal slice where the TFC is at its thinnest**  *Coronal PD and PD SPAIR* |  | 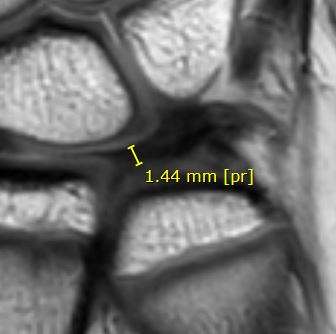  _ . _ _ mm at the thinnest point | | 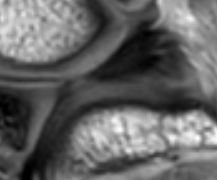0.00 mm when cannot be measured | |  |
| 1.4 | **Homogeneity**  *Coronal PD and PD SPAIR* | 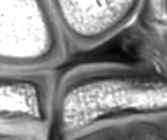  □ Homogeneous hypointens without increased signal | 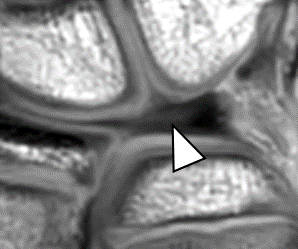  □ Diffuse increased signal not extending to the joint surface | | 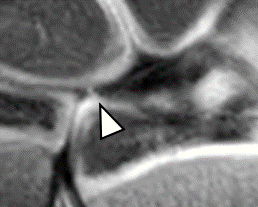  □ Linear vertical increased signal with disruption of the disc | | 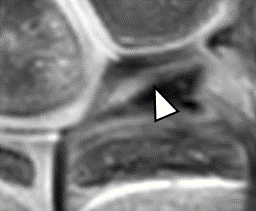  □ Other |

| **C** | **RADIOULNAR LIGAMENTS (RUL’s)** | |  | | | |
| --- | --- | --- | --- | --- | --- | --- |
| 1.1 | **Homogeneity dorsal RUL – sagittal**  *Sagittal PD* | 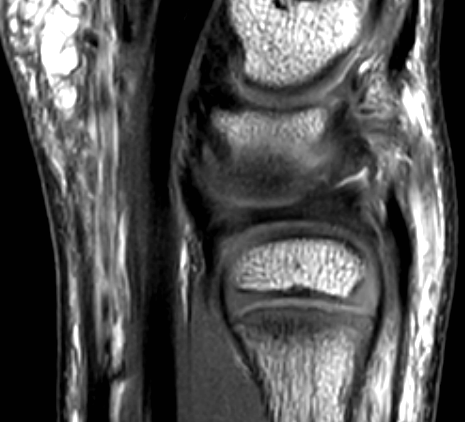  □ Continuous with TFC | | 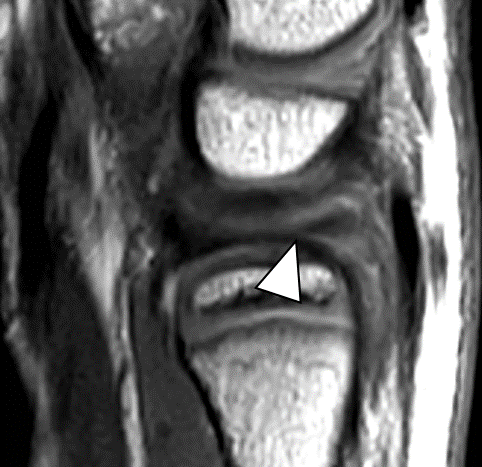  □ Not continuous with TFC | | □ Not able to assess |
| 1.2 | **Homogeneity volair RUL – sagittal**  *Sagittal PD* | 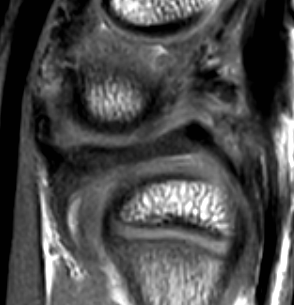  □ Continuous with TFC | | 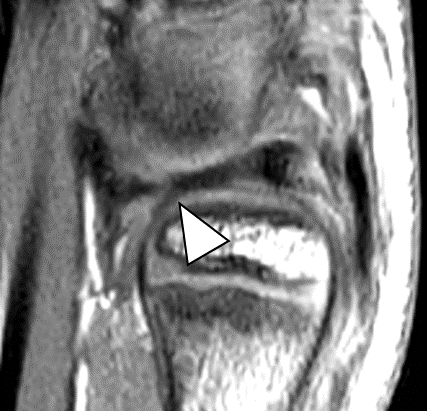  □ Not continuous with TFC | | □ Not able to assess |
| 1.3 | **Fiber continuity dorsal RUL – axial**  *Axial PD and T2 SPAIR* | 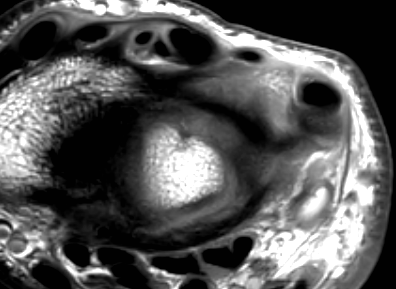  □ Continuous fibers | | 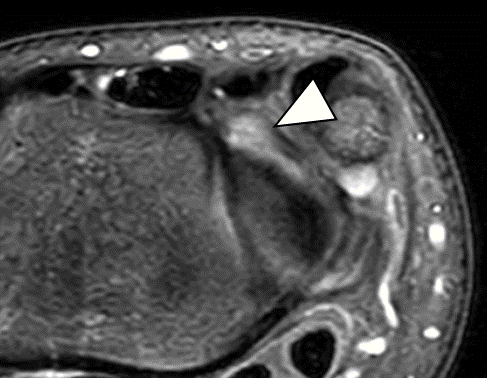  □ Fiber disruption | | □ Not able to assess |
| 1.4 | **Fiber continuity volar RUL – axial**  *Axial PD and T2 SPAIR* | 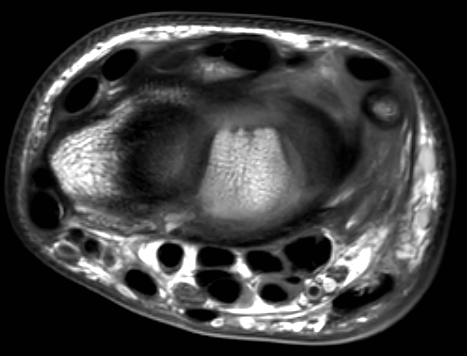  □ Continuous fibers | | □ Fiber disruption | | □ Not able to assess |
| **D** | **PROXIMAL (DEEP) AND DISTAL (SUPERFICIAL) LAMINA** | | | |  | |
| 1.1 | **Homogeneity proximal lamina**  *Coronal PD and PD SPAIR / sagittal PD* | 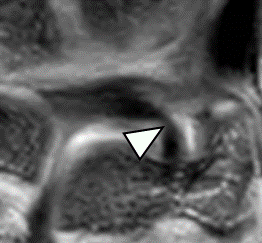  □ Homogeneous hypointens | | 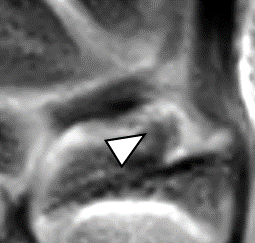  □ Diffuse lamination | | 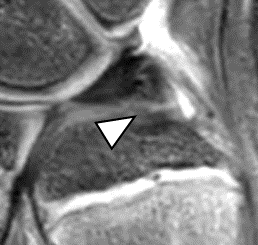  □ Not able to assess |

| 1.2 | **Homogeneity distal lamina**  *Coronal PD and PD SPAIR / sagittal PD* | 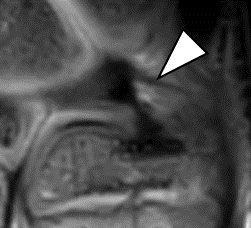  □ Homogeneous hypointens | | | 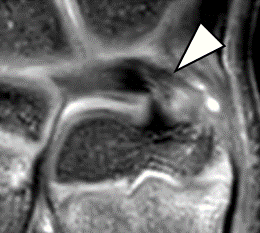  □ Diffuse lamination | | 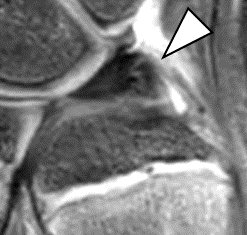  □ Not able to assess | |
| --- | --- | --- | --- | --- | --- | --- | --- | --- |
| **E** | **LIGAMENTUM SUBCRUENTUM** | | |  | | | | |
| 1 | **Visibility**  *Coronal PD SPAIR* |  | 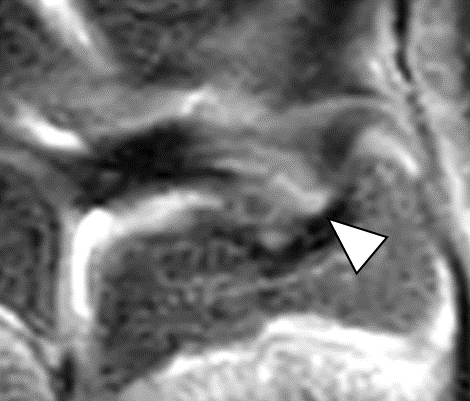□ Hyperintens signal between the proximal and distal lamina | | | 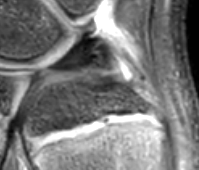  □ Not visible | |  |

| **F** | **PRESTYLOID RECESS** | | |  | | | | |
| --- | --- | --- | --- | --- | --- | --- | --- | --- |
| 1 | **Visibility**  *Coronal PD SPAIR* | 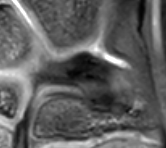  □ Tubular shaped | 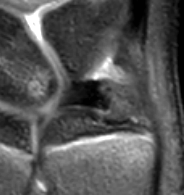  □ Conical shaped | | | 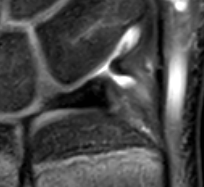  □ Saccular shaped | | 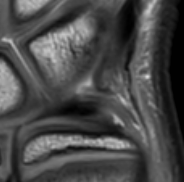  □ Not visible |
| **G** | **MENISCUS HOMOLOGUE** | | |  | | | | |
| 1 | **Visibility**  *Coronal PD en PD SPAIR* | 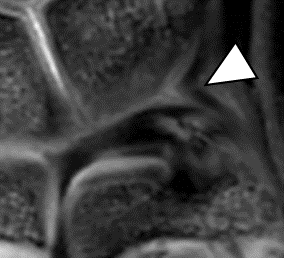  □ Clearly delineated hypointensity | | | 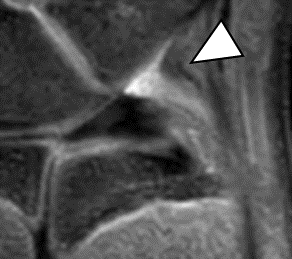  □ Diffuse hypointensity | | 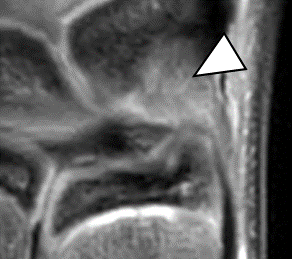  □ Not visible due to diffuse hyperintensity | |

| **H** | **EXTENSOR CARPI ULNARIS (ECU)** | | |  | | | | |
| --- | --- | --- | --- | --- | --- | --- | --- | --- |
| 1.1 | **ECU position in ulnar groove**  *Axial PD and T2 SPAIR* | 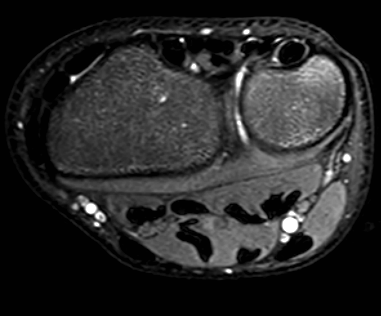  □ Completely within ECU groove | | | 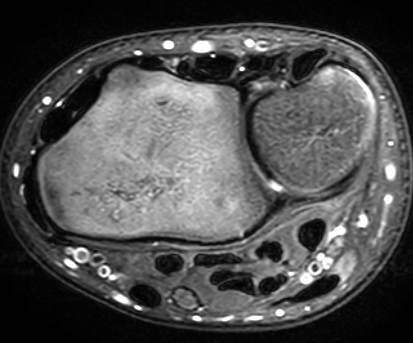  □ Partially within ECU groove | | 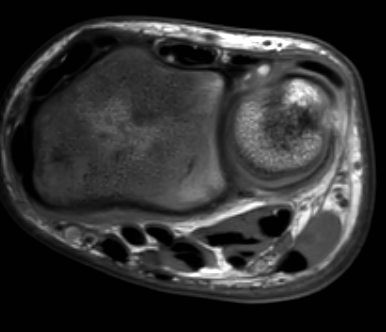  □ Completely outside ECU groove | |
| 1.2 | **Peritendinous signal intensity – from the ECU groove until distal extensor retinaculum**  *Axial T2 SPAIR* |  | 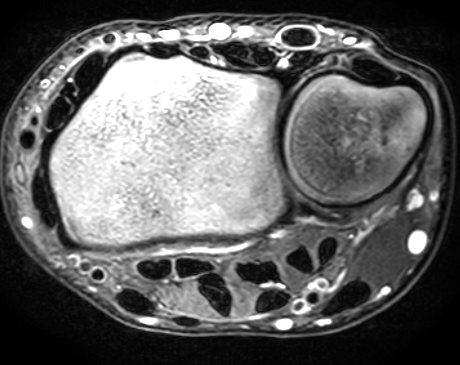  □ Hypointens or intermediate signal | | | 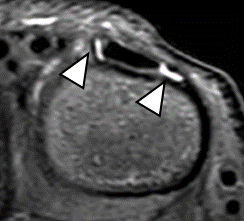  □ Focal increased signal | |  |

| 1.3 | **Intratendinous signal intensity – from the ECU groove until distal extensor retinaculum**  *Axial T2 SPAIR* |  | | 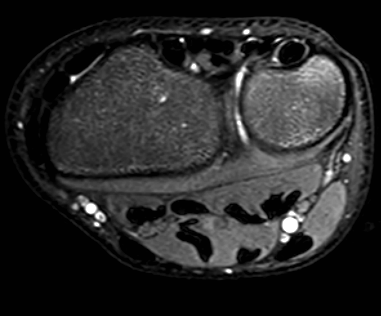  □ Homogeneous hypointens | 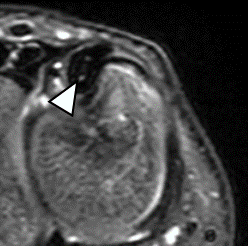  □ Focal or linear increased signal | |  |
| --- | --- | --- | --- | --- | --- | --- | --- |
| 1.4 | **Location increased signal**  *Axial T2 SPAIR* | □ Not applicable | □ Proximal from the styloid process | | □ At the styloid process level | □ Distal from the styloid process | |

| **I** | **DISTAL RADIOULNAR JOINT (DRUJ)** | |  | | |
| --- | --- | --- | --- | --- | --- |
| 1.1 | **Effusion radioulnar**  *Coronal PD SPAIR and axial T2 SPAIR* | 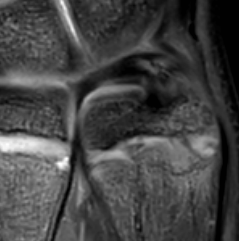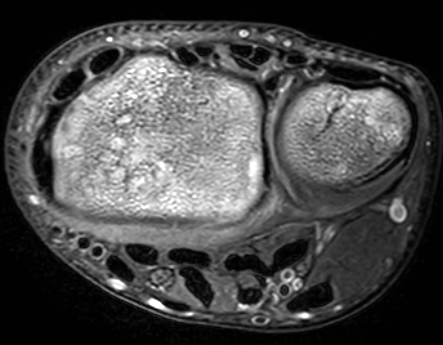  □ Absent | | 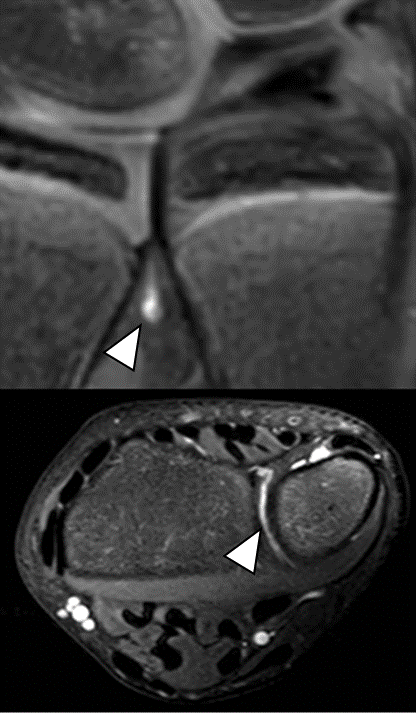  □ Small amount | 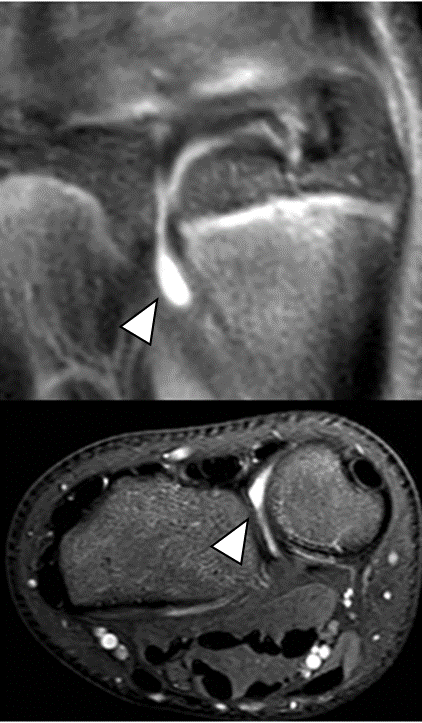  □ Substantial amount |

| 1.2 | **Cysts – ulnar sided wrist**  *Coronal PD SPAIR and axial T2 SPAIR* | 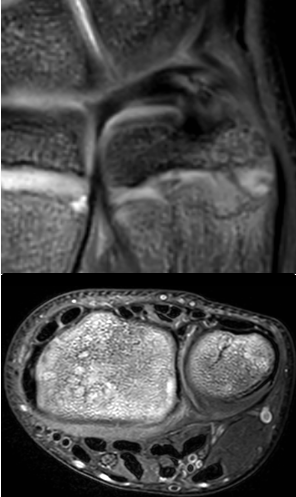  □ Absent | | 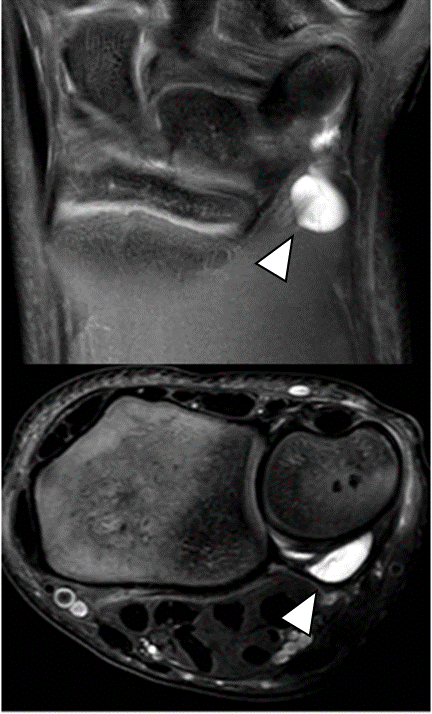  □ Volar | 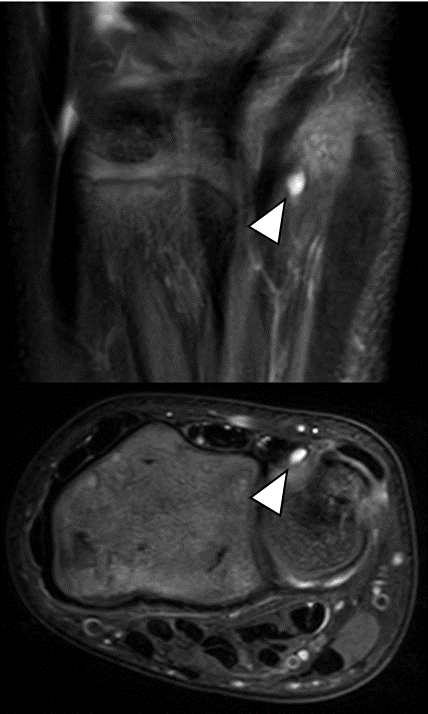  □ Dorsal |
| --- | --- | --- | --- | --- | --- |
| **J** | **PISOTRIQUETRAL JOINT (PTJ)** | |  | | |
| 1.1 | **PTJ effusion**  *Coronal PD SPAIR* | 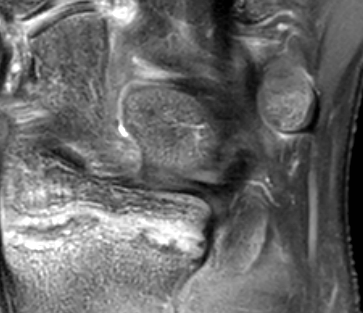  □ Absent | | 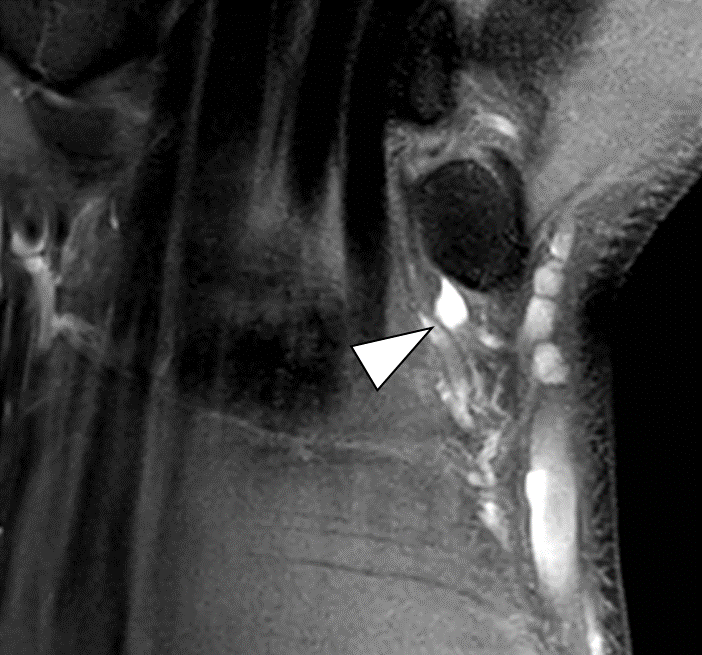  □ Small amount | 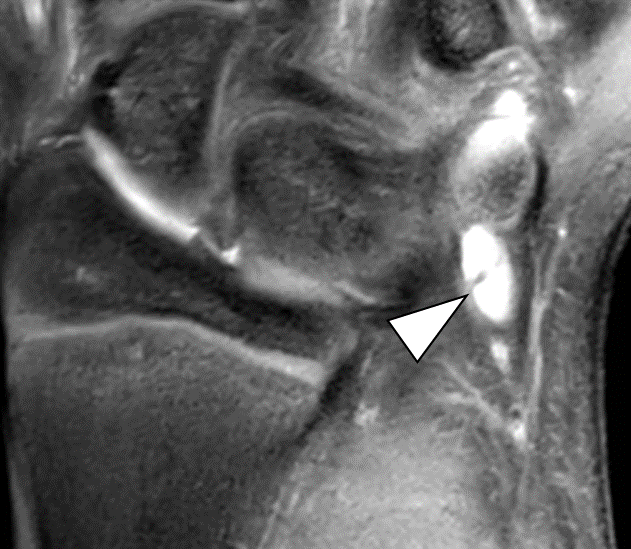  □ Substantial amount |

| **K** | **OTHER RELEVANT FINDINGS** |
| --- | --- |
|  | |
